# Ester alkaloids from *Cephalotaxus* interfere with the 2’3’-cGAMP-induced type I interferon pathway *in vitro*

**DOI:** 10.1371/journal.pone.0182701

**Published:** 2017-08-03

**Authors:** Gayoung Park, Sun Yeou Kim, Yoon-Jae Song

**Affiliations:** 1 Department of Life Science, Gachon University, Seongnam-Si, Kyeonggi-Do, Korea; 2 Laboratory of Pharmacognosy, College of Pharmacy, Gachon University, Incheon, Korea; Johns Hopkins School of Medicine, UNITED STATES

## Abstract

Dysregulated activation of the cyclic GMP-AMP synthase-stimulator of interferon genes (cGAS-STING) pathway by self-DNA contributes to interferonopathy and promotes autoimmune diseases. To identify potential suppressors of STING-induced type I interferon (IFN) induction, ethanol extracts of medicinal plants were screened for inhibitory activity against IFN-ß promoter activation. Notably, 70% ethanol extract of *Cephalotaxus koreana* specifically down-regulated STING-induced, but not TBK1- or IRF3-induced, IFN-ß promoter activity. The compounds exerting inhibitory activity specifically against STING-mediated IFN-ß promoter activation were identified as ester alkaloids isolated from the genus, *Cephalotaxus*, homoharringtonine and harringtonine. Furthermore, these two compounds inhibited 2’3’-cGAMP-induced IFN-stimulated gene expression and interaction between STING and TBK1. These suppressive effects were not observed with cephalotaxine devoid of the ester side-chain. Our data support the potential utility of homoharringtonine and harringtonine to treat STING-associated interferonopathy and autoimmune diseases.

## Introduction

The innate immune system plays an important role in detecting pathogen-derived nucleic acids. Pattern recognition receptors (PRR) recognize foreign pathogens, such as viruses, bacteria and parasites, and promote inflammation through activating various signaling cascades. Recognition of cytoplasmic DNA triggers the mechanisms of host defense and the production of type I interferon (IFN) [1, 2]. Among the network of signaling molecules, stimulator of interferon genes (STING), a resident protein of the endoplasmic reticulum (ER), is an essential adaptor protein that activates the type I IFN signaling pathway in response to cytosolic DNA. STING is mainly expressed in macrophages, dendritic cells, endothelial cells, T cells, and fibroblasts [3]. Following recognition of cytosolic DNA, STING relocalizes in the nucleus with TBK1 which promotes phosphorylation of IRF3 to induce type I IFN production [1, 4, 5]. The carboxyl terminus of STING is critical for activating TBK1 and recruiting IRF3 [4].

Cyclic di-GMP, a secondary messenger generated by bacteria, is reported to bind STING directly [6]. During recognition of intracellular DNA, cyclic guanosine monophosphate-adenosine monophosphate (cGAMP) synthase (cGAS) functions as a cytosolic DNA sensor activating reaction of GTP and ATP to form cGAMP, an endogenous secondary messenger that binds STING and stimulates the synthesis of type I IFN [7–9].

Inappropriate recognition of self-DNA leads to generation of autoantibodies and overproduction of cytokines including CXCL10, IFN-β, and TNF-α. Phagocytosed apoptotic and necrotic DNA that are incompletely digested due to deficiency of lysosome function dysregulate innate immune responses through a TLR-independent pathway and mediate interferonopathy and autoimmune diseases, such as systemic lupus erythematosus (SLE) and chronic polyarthritis (reviewed in [10]). In an earlier study, DNase II knock-out mice with markedly increased levels of IFN-ß and other cytokines died or exhibited signs of arthritis [11]. STING is proposed to be involved in over-production of inflammatory cytokines in response to self-DNA because cytokine levels and polyarthritis lesions are remarkably decreased in Dnase II and STING double knock-out mice [12]. Mutations in 3’ repair exonuclease1 (TREX1), previously known as Dnase III, also appear to trigger autoimmune diseases through interaction with STING. TREX1 degrades intracellular double-stranded DNA and negatively regulates STING-dependent innate immune responses [13]. Functional deficiency of TREX1 has been shown to cause accumulation of DNA and consistent activation of immune responses. Aicardi-Goutières syndrome (AGS) is one of the IFN-associated autoimmune diseases caused by mutation of the TREX1 gene [14, 15]. Autoimmune diseases caused by TREX1 mutations can be rescued by functional deficiency of IRF3 or type I IFN receptor (IFNR) [3]. Therefore, targeting STING to suppress the type I IFN response against self-DNA appears to present an effective strategy to treat autoimmune disease.

*In vitro* screening of medicinal plant extracts led to the identification of a 70% ethanol extract of *Cephalotaxus koreana* that specifically inhibits STING-induced, but not TBK1- or IRF3-induced IFN-β promoter activation. The effects of two major ester alkaloids isolated from the genus *Cephalotaxus* on STING-induced type I IFN signaling pathway were further investigated.

## Materials and methods

### Cell culture, plasmids, reagents and plant materials

Human embryonic kidney 293T (HEK293T) cells and human monocytic leukemia cell line THP-1 cells were obtained from Korean Cell Line Bank (Seoul, Korea). HEK293T cells were cultured in Dulbecco’s Modified Eagle Medium(DMEM) (Biowest, Nuaille, France) supplemented with 10% fetal bovine serum and 1% penicillin/streptomycin. THP-1 cells were cultured in RPMI 1640 (Thermo Fisher Scientific, Waltham, MA) supplemented with 10% fetal bovine serum, 1% penicillin/streptomycin and 0.05mM 2-mercaptoethanol. Human STING (hSTING), TBK1 and IRF3 were cloned into a pEF-based destination vector from the pENTR-hSTING, pENTR-hTBK1, and pENTR-hIRF3 plasmids, respectively, using LR clonase^™^ enzyme mix (Invitrogen, Carlsbad, CA). 2’3’-cGAMP was acquired from InvivoGen (San Diego, CA). Homoharringtonine was purchased from Sigma-Aldrich (St. Louis, MO) and harringtonine from Santa Cruz Biotechnology (Dallas, TX). Cephalotaxine was obtained from Glentham Life Sciences (Corsham, UK). OmicsFect^™^ *in vitro* transfection reagent (Omics Biotechnology, Taiwan) was employed for transient-transfection according to the manufacturer’s instructions. The plant material (*Cephalotaxus koreana*) used in this study was collected from Jeollanam-Do in Korea, and voucher specimens for the samples deposited at the herbarium of the Department of Biological Sciences at Sungkyunkwan University (specimen number 160628500). Extraction and fractionation of plant materials were performed in accordance with previously described procedures [16].

### Cell viability assay and luciferase reporter assay

Cell viability was analyzed using the Cell Titer-Glo luminescent assay (Promega, Madison, WI) according to the manufacturer’s instructions. The luciferase assay was performed as described previously [17].

### Quantitative RT-PCR

Total RNA was isolated using the Total RNA Prep kit (BioFact, Malaysia) and reverse transcribed into cDNA with the QuantiTect reverse transcription kit (Qiagen, Hilden, Germany) in keeping with the manufacturer’s guidelines. Real-time PCR reactions were carried out in a 20 μL reaction volume containing 1X HOT FIREPol^®^ EvaGreen^®^ PCR mix Plus (Solis BioDyne, Tartu, Estonia) with the following primers: IFNβ1, ATGACCAACAAGTGTCTCCTCC and GCTCATGGAAAGAGCTGTAGTG; CXCL10, TCCACGTGTTGAGATCATTGC and TCTTGATGGCCTTCGATTCTG; GAPDH, CATGAGAAGTATGACAACAGCCT and AGTCCTTCCACGATACCAAAGT.

### Immunoprecipitation and western blot

Cells were harvested and lysed in buffer containing 1% NP40, 150mM NaCl, 50mM Tris (pH 7.5), 1mM EDTA, 1mM PMSF, 50mM NaF, and protease inhibitor cocktail (Roche, Basel, Switzerland). Lysates were pre-cleared with A/G agarose beads (Santa Cruz Biotechnology) and incubated at 4°C over-night with anti-GST antibody. Next, lysates were washed three times with lysis buffer and subjected to western blot with the appropriate antibodies, as described previously [17]. Antibodies against STING and phospho-TBK1 were purchased from Cell Signaling Technology (Beverly, MA), antibodies against TBK1 and cGAS from Thermo Fisher Scientific (Waltham, MA) and Merck Millipore (Billerica, MA), and antibody against alpha-tubulin from Sigma-Aldrich (St. Louis, MO).

### Statistical analysis

Statistical analyses were carried out using JMP software (SAS Institute, Cary, NC). At least three independent experiments were performed, and error bars indicate as mean ± standard deviation. Significant difference between samples was determined by the *P* value of Student’s *t* test. IC_50_ values were determined by curve fitting using a four-parameter analysis.

## Results

### The *Cephalotaxus koreana* extract inhibits STING-induced IFN-β promoter activation in HEK293T cells

**Fig 1:**
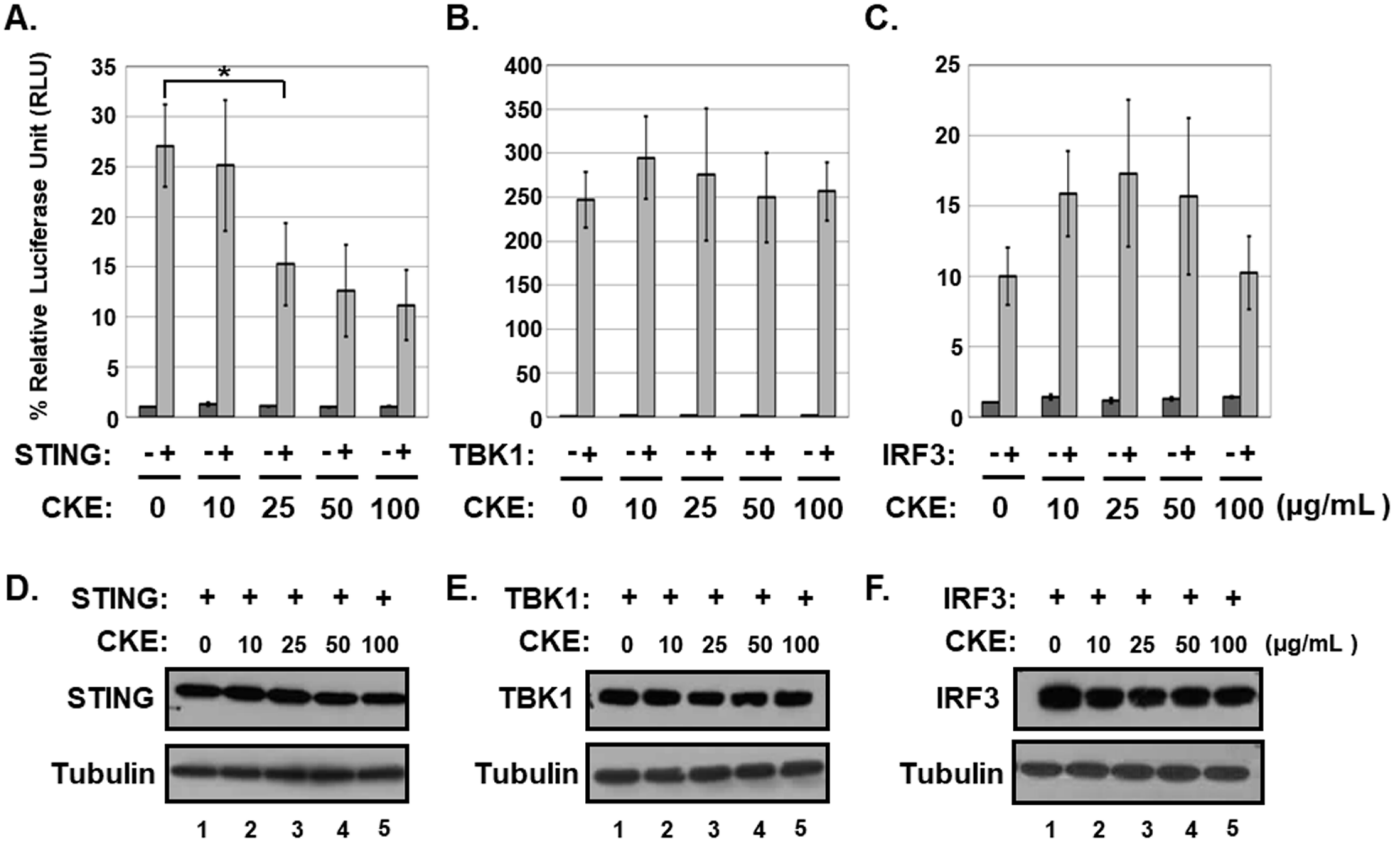
CKE inhibits STING-induced IFN-β promoter activation. HEK293T cells were co-transfected with control vector or vector expressing (A) hSTING, (B) TBK1 or (C) IRF3 plus IFN-β promoter-driven firefly luciferase and control *Renilla* luciferase plasmids. After transfection, cells were treated with DMSO or CKE at 10, 25, 50 or 100 μg/mL and luciferase activities measured using the dual-luciferase reporter assay system. Significant difference between samples was determined based on *P* values obtained from Student’s *t* test (* *P* < 0.05). (D, E and F) HEK293T cells were transfected with vector expressing (D) hSTING,(E) TBK1 or (F) IRF3 and treated with DMSO or CKE at 10, 25, 50 or 100 μg/mL. Equal amounts of cell extracts were subjected to western blot analysis with antibodies to STING, TBK1, IRF3 and tubulin.

Using the IFN-ß promoter-driven luciferase reporter, 70% ethanol extracts of 845 medicinal plants were screened for potential inhibitory effects on exogenous STING-induced IFN-β promoter activation in HEK293T cells which exhibit no detectable endogenous STING protein [18]. HEK293T cells were used for the screening to avoid additive effects of endogenous STING protein. Among the extracts tested, *Cephalotaxus koreana* extract (CKE) down-regulated STING-induced IFN-β promoter activation with an estimated 50% inhibitory concentration (IC_50_) of 35.13 ± 3.51 μg/mL (Fig 1A) but had no effects on TBK1- or IRF3-induced IFN-β promoter activation (Fig 1B and 1C). In addition, CEK did not attenuate levels of STING, TBK1 and IRF3 proteins (Fig 1D–1F). These data indicate that CKE contains active ingredients that specifically block the ability of STING without affecting protein levels of transgenes to activate the IFN-β promoter.

### HHT and HT inhibit STING-induced IFN-β promoter activation in HEK293T cells

**Fig 2:**
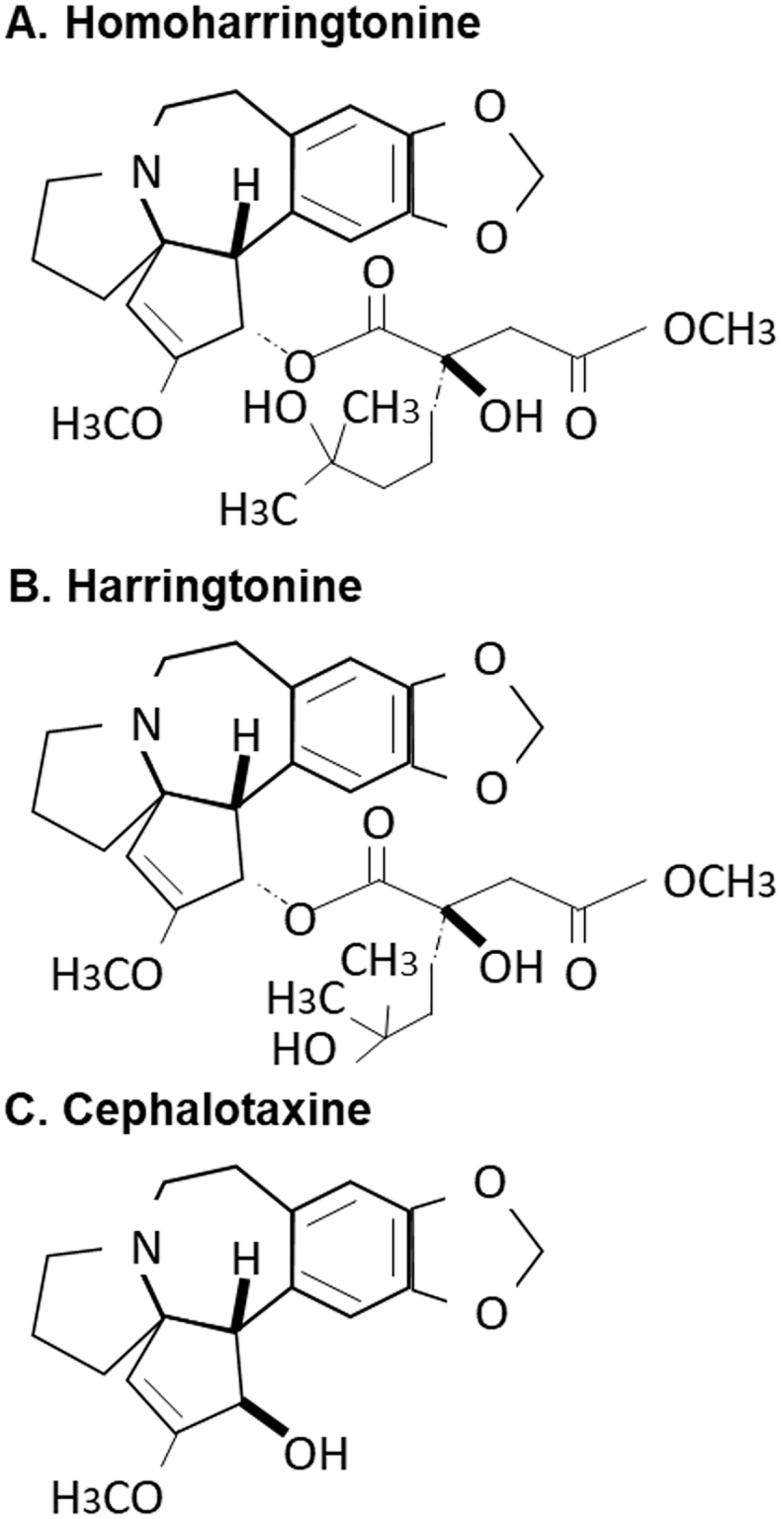
Structures of alkaloids used in this study. (A) homoharringtonine, (B) harringtonine and (C) cephalotaxine.

**Fig 3:**
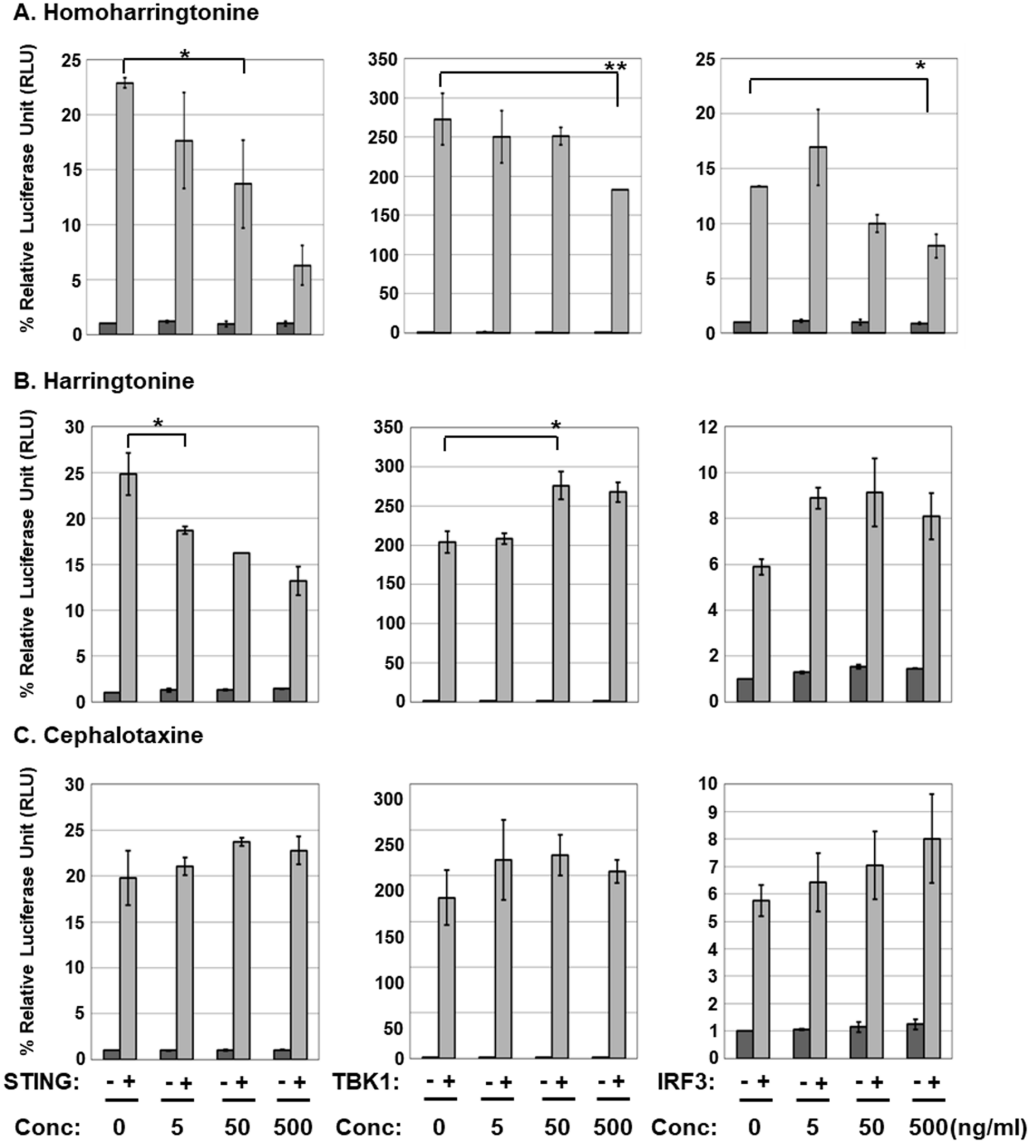
HHT and HT inhibit STING-induced IFNβ promoter activation. HEK293T cells were co-transfected with control vector or vector expressing hSTING, TBK1 or IRF3 plus IFN-β promoter-driven firefly luciferase and control *Renilla* luciferase plasmids. After transfection, cells were treated with (A) HHT, (B) HT or (C) CET at 0, 5, 50 or 500 ng/mL, and luciferase activity measured using the dual-luciferase reporter assay system. Significant difference between samples was determined based on *P* values obtained from Student’s *t* test (* *P* < 0.05, ** *P* < 0.01).

**Fig 4:**
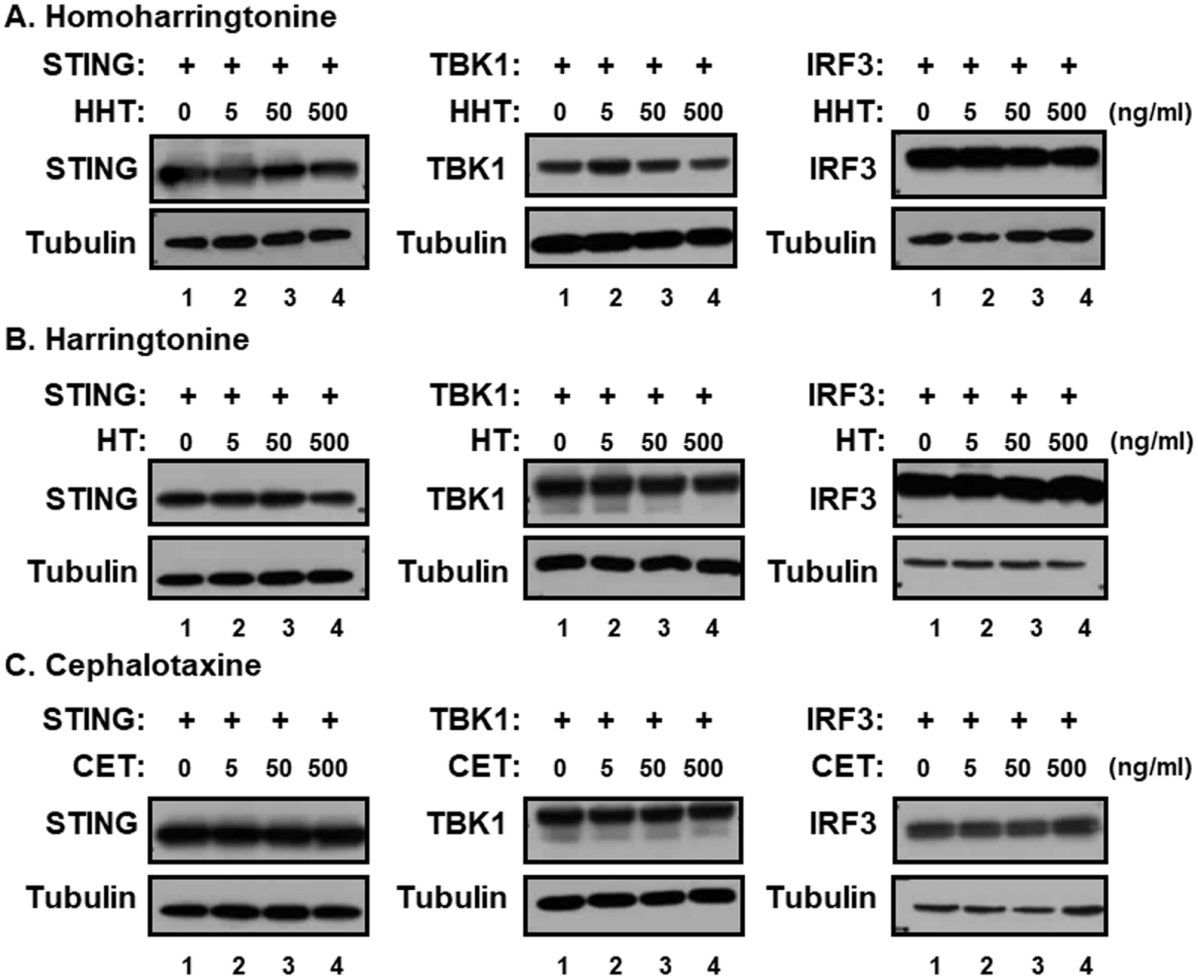
Effects of HHT, HT and CET on levels of STING, TBK1 and IRF3 proteins. HEK293T cells were transfected with vector expressing hSTING, TBK1 or IRF3 and treated with (A) HHT, (B) HT or (C) CET at 0, 5, 50 or 500 ng/mL. Equal amounts of cell extracts were subjected to western blot analysis with antibodies to STING, TBK1, IRF3 and tubulin.

The genus *Cephalotaxus* including the species *Cephalotaxus koreana*, contains alkaloids, such as cephalotaxine (CET) and its esters homoharringtonine (HHT) and harringtonine (HT) (Fig 2) [19]. In view of the inhibitory effect of CKE on STING-induced IFN-β promoter activation, the effects of HHT, HT and CET were further investigated. CET is biologically inactive, but its ester derivatives have antileukemic activity [20]. Interestingly, HHT and HT, but not CET, suppressed STING-mediated IFN-β promoter activation in a dose-dependent manner with IC_50_ values of 0.267±0.06 and 0.663±0.11 μg/mL, respectively, while exerting no significant inhibitory effects against TBK1- and IRF3-induced IFN-β promoter activation (Fig 3). At 500 ng/mL, HHT reduced TBK1- and IRF3-induced IFN-β promoter activation by 27 and 38%, respectively, possible due to slight decrease in TBK1 expression (Fig 4A) and/or adverse effects mediated by high concentrations of HHT. Nevertheless, HHT, HT and CET exerted no significant effects on protein levels of STING, TBK1 and IRF3 (Fig 4). Since CET had no effect on STING-, TBK1- or IRF3-induced IFN-β promoter activation, these data indicate that the ester side-chains of HHT and HT contribute significantly to this suppressor activity.

**Fig 5:**
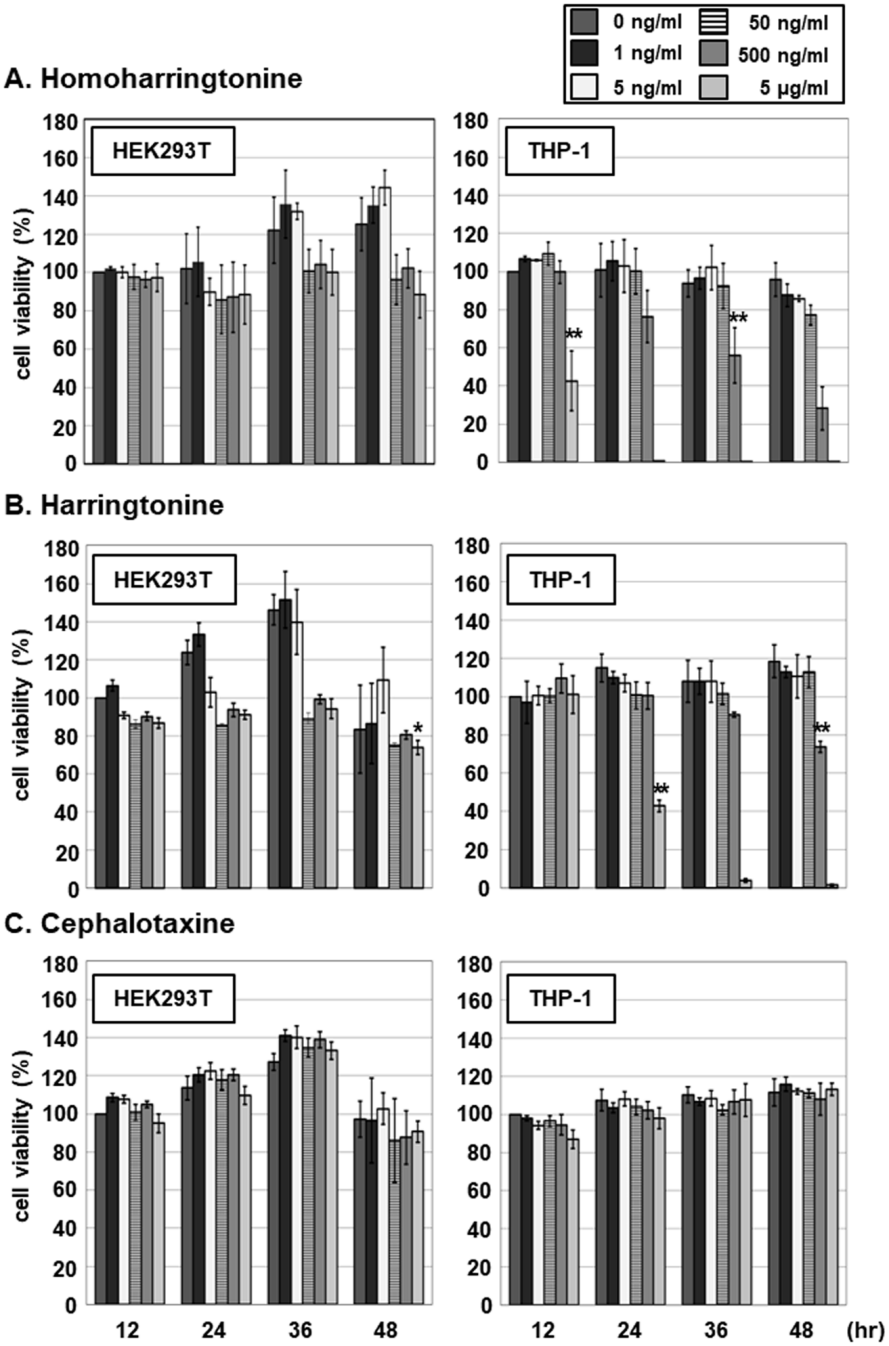
Cytotoxic effects of HHT, HT and CET on HEK293T and THP-1 cells. HEK293T and THP-1 cells were treated with HHT, HT, or CET at 0, 1, 5, 50, 500 or 5000 ng/mL. Cell viability was analyzed at 12, 24, 36 and 48 h after treatment using the Cell Titer-Glo luminescent cell viability assay. Significant reduction in cell viability compared to the control was determined based on *P* values obtained from Student’s *t* test (* *P* < 0.05, ** *P* < 0.01).

To determine whether the inhibitory activities are due to cytotoxicity, the effects of HHT, HT and CET on viability of HEK293T and THP-1 cells were further evaluated. HEK293T and THP-1 cells were treated with 0, 1, 5, 50 or 500 ng/mL HTT, HT and CET, followed by assessment of cell viability by measuring cellular ATP levels using the CellTiter-Glo assay at 12, 24, 36 or 48 h after treatment (Fig 5). Within 48 h, HHT exerted no significant cytotoxic effects against HEK293T cells at concentrations up to 500 ng/mL (Fig 5A). At 500 ng/mL, HT exhibited cytotoxic effect as evident from the 25% reduction in ATP level relative to that in cells in the 0 μg/ml treatment group at 12 h (Fig 5B). In comparison, THP-1 cell viability was reduced by 42% at 36 h after HHT treatment at 500ng/mL (Fig 5A) while HT did not exert a cytotoxic effect. At 48 h after treatment, 500ng/mL HT reduced the viability of THP-1 cells by 23% (Fig 5B). No cytotoxic effects of CET against HEK293T and THP-1 cells were observed (Fig 5C). The IC_50_ values of HHT and HT for THP-1 cell viability at 48 h were determined as 0.306.5 ± 0.07 and 1.579.2 ± 0.16 μg/mL, respectively. Since the viability of HEK293T was not affected by HHT and HT, we propose that the inhibitory effects of HHT and HT on STING-induced IFN-β promoter activation and ISG expression in this cell line are not mediated via cytotoxicity.

### HHT and HT inhibit 2’3’-cGAMP-induced induction of IFN-stimulated gene expression

**Fig 6:**
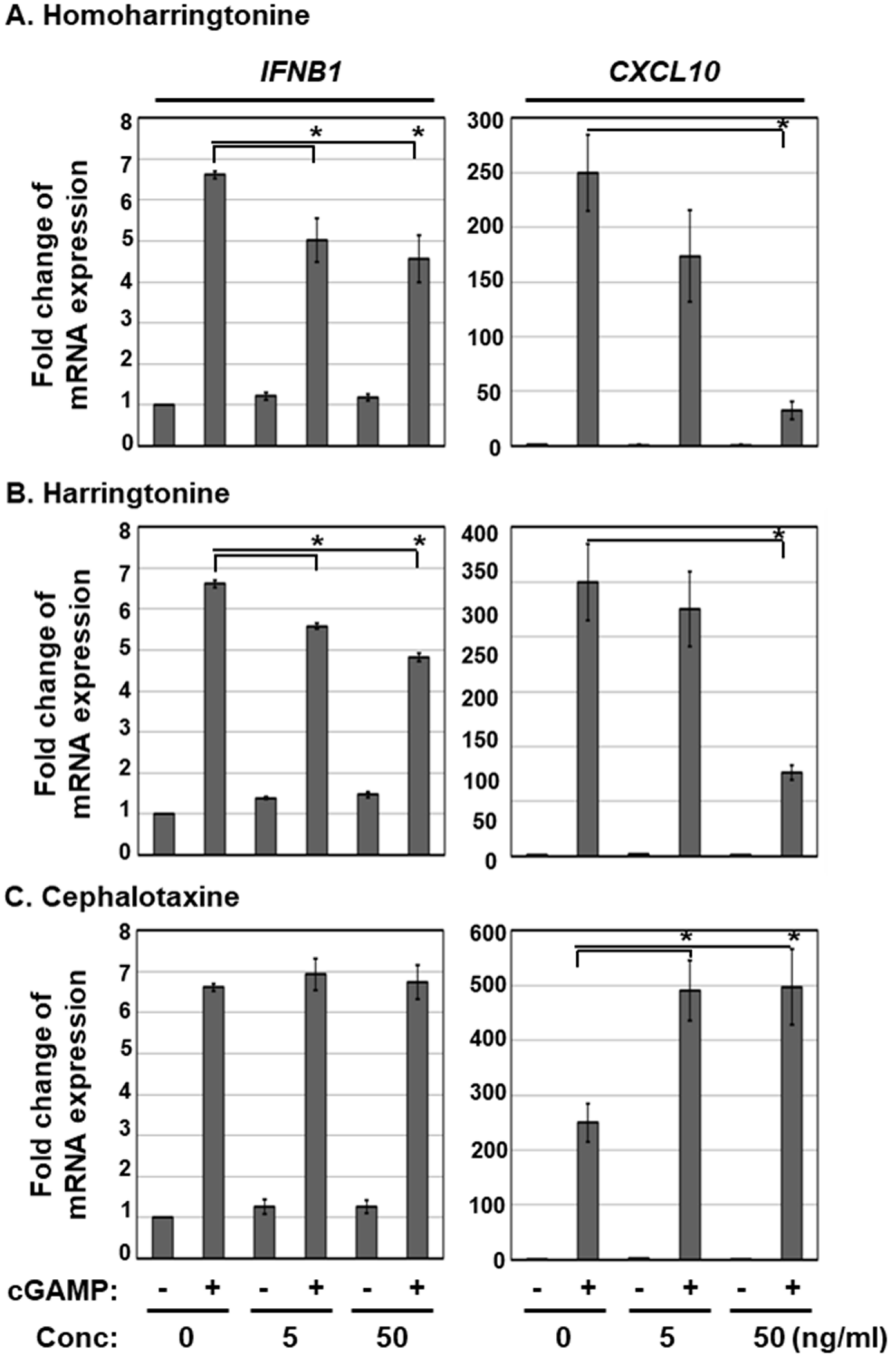
HHT and HT inhibit cGAMP-induced ISG expression. THP-1 cells were pre-treated with HHT, HT, or CET at 5 and 50 ng/mL for 18 h and transfected with 2’3’-cGAMP. At 6 h after transfection, the relative levels of IFNB1 and CXCL10 transcripts were determined using qRT-PCR. Significant difference between samples was determined based on *P* values obtained from Student’s *t* test (* *P* < 0.05).

To further investigate the effects of HTT and HT on STING-induced type I IFN pathway, THP-1 cells were pre-treated with HHT, HT or CET at the indicated concentrations and transfected with a STING agonist, 2’3’-cGAMP. At 6 h after 2’3’-cGAMP transfection, transcript levels of IFN-stimulated genes (ISG) were determined via qRT-PCR (Fig 6). We detected a 6.6- and 249-fold increase in *IFNβ1* and *CXCL10* transcript expression, respectively, which was significantly reduced in the HHT or HT treatment groups in a dose-dependent manner (Fig 6A and 6B). In contrast, CET had no effect on 2’3’-cGAMP-induced *IFNβ1* and *CXCL10* expression (Fig 6C). Interestingly, co-treatment with CET and 2’3’-cGAMP led to a synergistic increase in *CXCL10* transcript expression (Fig 6C, right panel). These findings clearly indicate that HHT and HT inhibit 2’3’-cGAMP-induced expression of ISGs.

### HHT and HT interfere with interactions between STING and TBK1 and consequent STING-induced TBK1 activation

**Fig 7:**
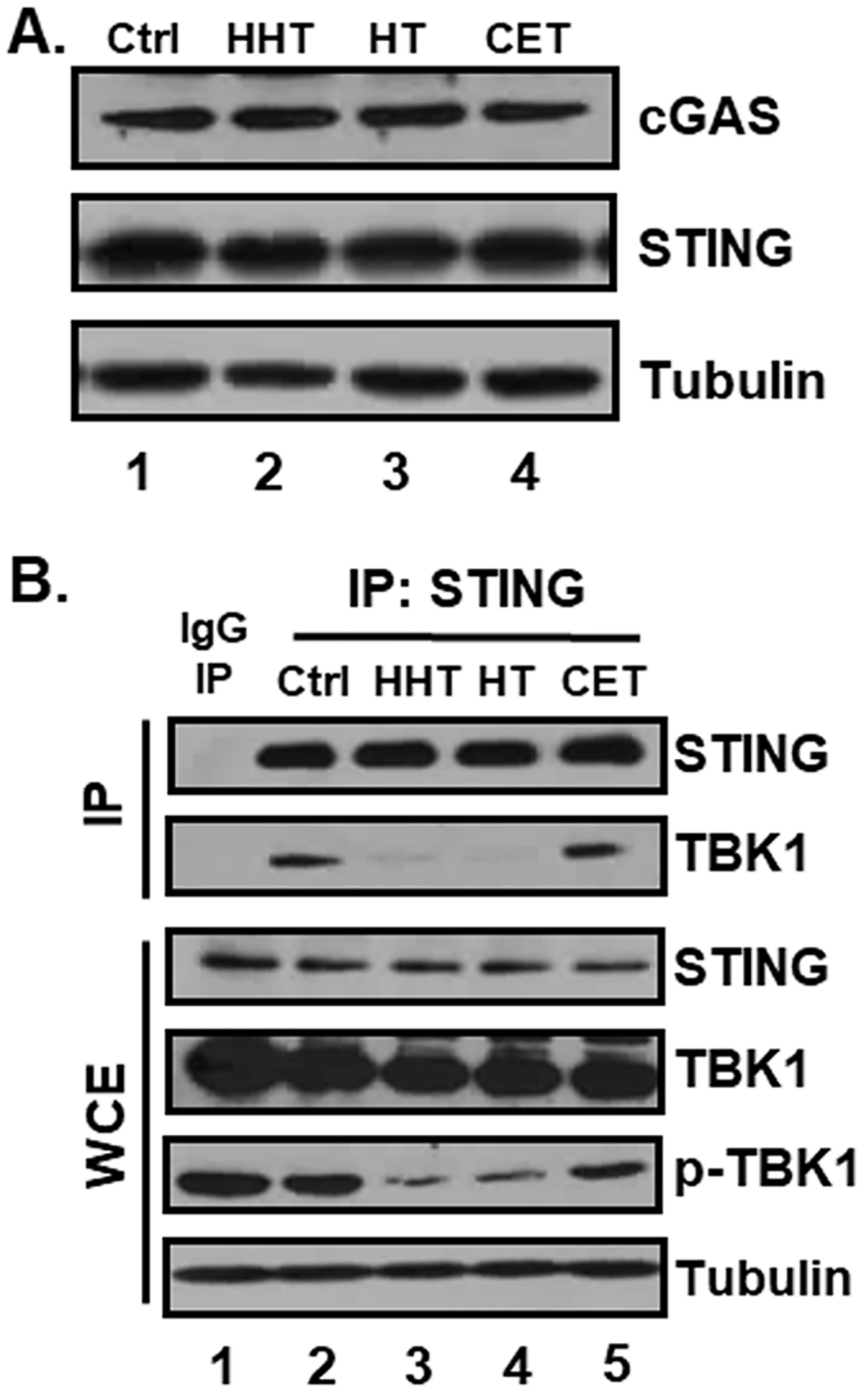
HHT and HT inhibit interactions between STING and TBK1. (A) THP-1 cells were treated with HHT, HT, or CET at 50 ng/mL for 18 h, and equal amounts of cell lysates were subjected to western blot with antibodies against cGAS, STING and tubulin. (B) HEK293T cells were transfected with a vector expressing hSTING. After transfection, cells were pre-treated with DMSO, HHT, HT or CET for 16 h and transfected with 2’3’-cGAMP. At 6 h after transfection, cell lysates were immunoprecipitated with either anti-IgG or anti-STING antibody, and STING immunoprecipitates (IP) and whole cell extracts (WCE) were subjected to western blot analysis of of STING, TBK1, phospho-TBK1 and tubulin.

Although HHT and HT are reported to inhibit translation of short-lived proteins including c-Myc, Mcl-1 and cyclin D1 [19], in our experiment, cGAS and STING protein levels were not affected by treatment with the two ester alkaloids (Fig 7A). We further determined the effects of HHT, HT and CET on STING and TBK1 interactions. In HEK293T cells expressing ectopic STING protein, 2’3’-cGAMP treatment induced the interaction between STING and TBK1 and, in turn, phosphorylation of TBK1, an indicator of TBK1 activation (Fig 7B, lane 2). However, in cells treated with HHT or HT, interactions between STING and TBK1 and TBK1 phosphorylation were significantly reduced (Fig 7B, lanes 3 and 4). On the other hand, CET had no effect on 2’3’-cGAMP-induced binding between STING and TBK1 and TBK1 phosphorylation (Fig 7B, lane 5). These data indicate that HHT and HT inhibit the 2’3’-cGAMP-induced signaling pathway by interfering with interactions between STING and TBK1.

## Discussion

Dysregulated turnover and accumulation of self-DNA in the cytosol constitutively activates the cGAS-STING pathway to produce type I IFNs. Hyperproduction of type I IFNs and pro-inflammatory cytokines contributes to the pathogenesis of autoinflammatory diseases, such as AGS and SLE (reviewed in [12]). Thus, molecules that specifically inhibit the function of STING may provide effective novel drug candidates to treat autoinflammatory diseases caused by inappropriate sensing of self-DNA.

Screening of medicinal plant extracts facilitated the identification of CKE that specifically inhibits STING-induced, but not TBK1- or IRF3-induced, IFN-β promoter activation. The genus *Cephalotaxus* including *Cephalotaxus koreana* is distributed in China, eastern India, Thailand, Japan and Korea and used in traditional Chinese medicine to treat inflammation, infection and cancers [19]. HHT, HT and CET are alkaloid constituents of the genus *Cephalotaxus*, and the concentrations of HHT, HT and CET in *Cephalotaxus koreana* were previously reported [21]. In our experiments, HHT and HT, but not CET, specifically inhibited STING-induced IFN-ß promoter activation and cGAMP-induced expression of ISGs. The tetracyclic alcohol, CET, is a major component of *Cephalotaxus* and reported to be biologically inactive [22]. On the other hand, HHT and HT, which are classified according to the modification of the esters of CET, exert anti-inflammatory and anti-tumor effects [19]. In HHT, 4-methyl-2-hydroxy-4-methylpentyl butanedioate is replaced with a succinic acid ester of cephalotaxine [23]. HHT and HT are homologs with differences only in the ester group (Fig 2) [22, 24] that have been widely used to treat various leukemia types, such as chronic myeloid leukemia (CML), acute myeloid leukemia (AML) and myelodysplastic syndrome (MDS) [19]. Semisynthetic HHT, also known as omacetaxine mepesuccinate, is produced by direct esterification of CET and approved by the Food and Drug Administration (FDA) for treatment of CML resistant to tyrosine kinase inhibitors [25, 26].

The existence of an ester side-chain at C-3 is a key factor underlying the antitumor activities of HHT and HT. The ester side-chain is proposed to contribute to the inhibitory effects of HHT and HT on STING activity, in view of the finding that CET has no effect on STING-induced IFN-ß promoter activation and cGAMP-induced expression of ISGs. Both HHT and HT suppress the synthesis of short-lived proteins by inhibiting the elongation phase of translation [27]. HHT is additionally reported to suppress the STAT3 signaling pathway via up-regulation of IL-6 [28]. Data from the current study indicate that HHT and HT exert no adverse effects on protein levels of cGAS and STING, but interfere with interactions between STING and TBK1 and, in turn, subsequent activation of IRF3. Structure-function studies are necessary to provide insights into the mechanisms by which these ester alkaloids block STING-TBK1 interactions. In addition, *in vivo* and clinical studies are a focus of further investigations to validate the potential application of HHT and HT in treating interferonopathy and autoimmune diseases caused by dysregulation of the cGAS-STING pathway.

## Abbreviations

CET: cephalotaxine
cGAMP: Cyclic [G(2’5’)pA(3’5’)p]
HHT: homoharringtonine
HT: harringtonine
IFN: interferon
STING: stimulator of interferon genes
